# Defining the molecular pathologies in cloaca malformation: similarities between mouse and human

**DOI:** 10.1242/dmm.014530

**Published:** 2014-02-13

**Authors:** Laura A. Runck, Anna Method, Andrea Bischoff, Marc Levitt, Alberto Peña, Margaret H. Collins, Anita Gupta, Shiva Shanmukhappa, James M. Wells, Géraldine Guasch

**Affiliations:** 1 Division of Developmental Biology, Cincinnati Children’s Hospital Medical Center, 3333 Burnet Avenue, Cincinnati, OH 45229, USA.; 2 Division of Pediatric General and Thoracic Surgery, Colorectal Center, Cincinnati Children’s Hospital Medical Center, 3333 Burnet Avenue, Cincinnati, OH 45229, USA.; 3 Division of Pediatric Pathology, Cincinnati Children’s Hospital Medical Center, 3333 Burnet Avenue, Cincinnati, OH 45229, USA.; 4 Division of Endocrinology, Cincinnati Children’s Hospital Medical Center, 3333 Burnet Avenue, Cincinnati, OH 45229, USA.

**Keywords:** Anorectal malformation, Cloaca, Patterning, Epithelial differentiation, Sonic hedgehog

## Abstract

Anorectal malformations are congenital anomalies that form a spectrum of disorders, from the most benign type with excellent functional prognosis, to very complex, such as cloaca malformation in females in which the rectum, vagina and urethra fail to develop separately and instead drain via a single common channel into the perineum. The severity of this phenotype suggests that the defect occurs in the early stages of embryonic development of the organs derived from the cloaca. Owing to the inability to directly investigate human embryonic cloaca development, current research has relied on the use of mouse models of anorectal malformations. However, even studies of mouse embryos lack analysis of the earliest stages of cloaca patterning and morphogenesis. Here we compared human and mouse cloaca development and retrospectively identified that early mis-patterning of the embryonic cloaca might underlie the most severe forms of anorectal malformation in humans. In mouse, we identified that defective sonic hedgehog (Shh) signaling results in early dorsal-ventral epithelial abnormalities prior to the reported defects in septation. This is manifested by the absence of Sox2 and aberrant expression of keratins in the embryonic cloaca of *Shh* knockout mice. *Shh* knockout embryos additionally develop a hypervascular stroma, which is defective in BMP signaling. These epithelial and stromal defects persist later, creating an indeterminate epithelium with molecular alterations in the common channel. We then used these animals to perform a broad comparison with patients with mild-to-severe forms of anorectal malformations including cloaca malformation. We found striking parallels with the Shh mouse model, including nearly identical defective molecular identity of the epithelium and surrounding stroma. Our work strongly suggests that early embryonic cloacal epithelial differentiation defects might be the underlying cause of severe forms of anorectal malformations in humans. Moreover, deranged Shh and BMP signaling is correlated with severe anorectal malformations in both mouse and humans.

## INTRODUCTION

Anorectal malformations are congenital anomalies that encompass a wide spectrum of diseases and occur in ~1 in 5000 live births (Levitt and Peña, 2007). The anorectal and urogenital systems arise from a common transient embryonic structure called the cloaca that exists from the fourth week of intrauterine development in humans (Fritsch et al., 2007; Kluth, 2010) and between days 10.5–12.5 post-fertilization in mice (Seifert et al., 2008). By the sixth week in humans the embryonic cloaca is divided, resulting in a ventral urogenital sinus and a separate dorsal hindgut. By the twelfth week, the anal canal, vaginal and urethral openings are established. Defective development of the embryonic cloaca results in anorectal and urogenital malformations, which are some of the most severe congenital anomalies encountered in children. The most severe form in females is termed cloaca malformation (Warne et al., 2011) and these present as a wide spectrum of defects. Generally, cloaca malformation is characterized by the junction of urethra, vagina and rectum forming a common channel that opens as a single orifice. The length of the common channel can vary from 1 to 10 cm and has important clinic implications. The longer the common channel (>3 cm), the higher the incidence of associated anomalies as well as there being poor prognosis for bowel and urinary control. The surgical reconstruction also represents a technical challenge for the surgeon (Levitt and Peña, 2010).

The molecular and morphogenetic mechanisms that pattern and subdivide the embryonic cloaca into these different epithelial structures are still unclear. Most of the recent attempts at elucidating cloacal embryogenesis relied on the use of aborted or non-viable fetuses or animal models (Penington and Hutson, 2002). Studies of the sequence of embryonic cloacal development in humans have been fraught with conflicting results on whether the lateral plates fuse to compartmentalize the embryonic cloaca or rather that the separation into the three structures (vagina, rectum and urethra) results from a differential rate of growth between the cephalic and caudal components of the embryonic cloaca (Nievelstein et al., 1998; van der Putte, 2005; Fritsch et al., 2007; van der Putte, 2009). Works in zebrafish have brought insight into defects in cloaca malformation by showing a migratory defect of cells that form the cloaca opening, preventing programmed cell death of ectodermal cells (Slanchev et al., 2011). In this model, proper mitotic spindle orientation seems to be required for cloaca morphogenesis (Bubenshchikova et al., 2012).

Another important consideration has been the crucial role of the interactions between the epithelial and stromal components in the differentiation process (Roberts et al., 1998; van der Putte, 2005; Fritsch et al., 2007). During the early stages of embryonic cloacal development, the dominant events are massive epithelial apoptosis of the urorectal septum, fusion of the epithelial walls of the cloaca and the simultaneous active growth of the mesenchyme within the urorectal septum (Qi et al., 2000). Additionally, recent work has shown the crucial role of the intrinsic regulators of the mesenchyme surrounding the embryonic cloaca, *Six1* and *Eya1*, during genitourinary tract formation, including in cloaca septation (Wang et al., 2011). Therefore, the mesenchyme surrounding the embryonic cloaca is crucial for that process.

**TRANSLATIONAL IMPACT**

**Clinical issue**

The anorectal and urogenital systems arise from a common embryonic structure termed the cloaca. Defective cloacal development and the resulting anorectal and urogenital malformations are among the most severe birth defects. The most severe form of anorectal malformation in females – cloaca – is characterized by failed septation of the rectum, vagina and urethra, resulting in a common channel and a single orifice. Patients with cloaca malformation have an increased risk of urological renal impairment and life-threatening chronic urinary tract infections. Surgical reconstruction in cloaca is a challenge and 25% of the cases need vaginal replacement with bowel. Unfortunately, the neovagina epithelium created during this process is susceptible to tumor formation.

**Results**

Because it is difficult to follow the embryonic stages of cloaca development in humans, mouse models are a valuable tool for determining the molecular mechanisms involved in cloaca malformation. In this study, the authors utilize the *Shh* knockout mouse model, which develops a cloaca malformation, to determine whether cloaca results from defective differentiation of the embryonic cloacal epithelium. The authors report that, before septation, when the embryonic cloaca is still a cavity, the epithelium in control embryos already contains distinct regions that express keratins and transcription factors such as Sox2 and CDX2, which define the nature of the epithelium. By contrast, differentiation of the cloacal epithelium in *Shh* knockout mice is abnormal, a change that is manifested by the absence of Sox2 and aberrant keratin expression. Moreover, the cloacal epithelium in *Shh* knockout mice is surrounded by a hypervascular stroma that is defective in BMP signaling. These epithelial and stromal defects persist and subsequently create an indeterminate epithelium in the common channel. Notably, the authors report that Shh expression is absent or decreased in tissues collected from patients with cloaca malformation and show that the defective molecular patterning of the epithelium and surrounding stroma in these tissues parallels that in *Shh* knockout mice.

**Implications and future directions**

These findings suggest that defects in epithelial differentiation, and altered Shh and BMP signaling, occur early in cloaca development and then persist. They provide further evidence that, in *Shh* knockout mice, the epithelium from the cloaca malformation does not result in the persistence of the normal embryonic cloaca epithelium. The knowledge that proper differentiation of the cloaca epithelium at early developmental stages is necessary for the development of a separate urogenital and anorectal system provides a molecular basis for tissue engineering efforts that could aid in better surgical reconstruction.

Paracrine factors that mediate cross-talk between the mesenchyme and epithelium play important roles in the development of cloacal derivatives. For example, BMP7 from the urorectal mesenchyme plays a role in cloacal partitioning and reorganization of the endodermal epithelium (Wu et al., 2009; Xu et al., 2012). Cloacal septation depends on epithelial-to-mesenchymal signaling mediated by sonic hedgehog (*Shh*) from the cloacal endoderm (Haraguchi et al., 2001; Mo et al., 2001; Perriton et al., 2002; Haraguchi et al., 2007; Lin et al., 2009; Seifert et al., 2009; Seifert et al., 2010). Mutations in Shh or its downstream mediators, Gli2 and Gli3, result in the different forms of anorectal malformations (Kimmel et al., 2000). In the absence of Gli2, the most posterior end of the hindgut fails to differentiate into anorectum, whereas epithelial differentiation of the urogenital tract remains intact (Mo et al., 2001). Moreover, gene dosage of *Gli2* and *Gli3* modulates the severity of the malformation, suggesting that a precise degree of Shh signaling is required for the normal development of rectum and anus. In fact, mice with mutations in Shh signaling pathways recapitulate the whole spectrum of anorectal malformations that are seen in humans (Kim et al., 2001; Mo et al., 2001; Haraguchi et al., 2007; Lin et al., 2009; Seifert et al., 2009), implicating this pathway in the pathogenesis of cloaca anomalies. However, the molecular pathways that have been identified in mouse cloacal development (BMP, Shh) have not been implicated in anorectal malformations in humans, probably owing to a lack of available tissues.

In this study, we analyzed the nature of the epithelial and the stromal defects found in the cloaca of *Shh* knockout mice and compared these with surgical tissues from human cloaca patients to determine whether defects in Shh signaling correlate with the pathology of the disease in humans. We identified that the cloacal epithelium in *Shh* knockout mice has molecular defects as early as embryonic day 11.5 (E11.5), before septation. Moreover, there was reduced BMP signaling and hypervascularity in the stroma. Those abnormalities persisted later during development, resulting in the formation of an indeterminate epithelium with molecular alteration in the common channel. Interestingly, the epithelium found in the common channel of cloaca patients, which, on histology, resembled squamous mucosa, colonic mucosa, transitional epithelium, urothelium and urethral epithelium, was also composed of an indeterminate type of epithelium with molecular alterations and a decrease in Shh expression, suggesting that the molecular aberrations might not necessarily correlate with the histology phenotype. This similarity between the *Shh* knockout mouse model and the human cloaca malformation samples is also seen by the presence of a hypervascular stroma surrounding the human common channel, defective for BMP signaling. These results suggest that the more severe forms of cloacal defects in humans might also trace back to an early defect in cloacal development prior to septation.

## RESULTS

### Shh deficiency in mice affects the molecular identity of the embryonic cloaca epithelium before septation

**Fig. 1.**
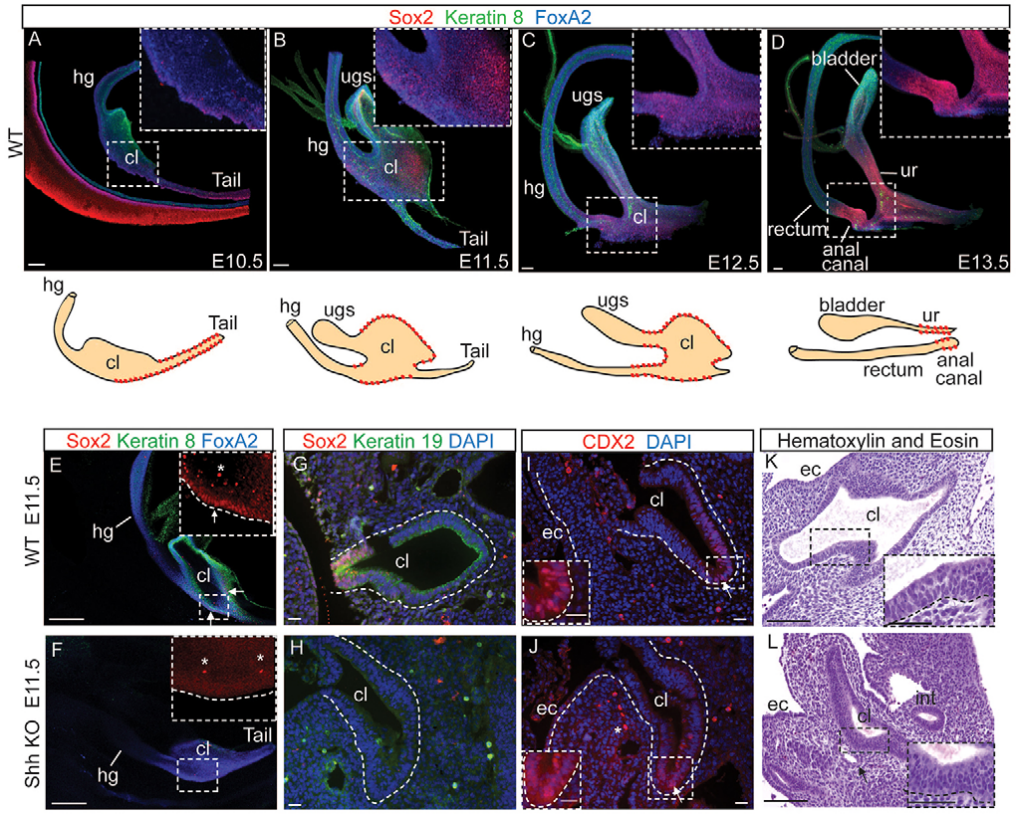
**Molecular defects in the cloaca epithelium of *Shh* knockout embryos.** (A–D) Whole-mount immunofluorescence showing early patterning of the wild-type (WT) cloaca epithelium in E10.5 (A), E11.5 (B), E12.5 (C) and E13.5 (D) embryos stained with Sox2 (red), keratin 8 (green) and FoxA2 (blue). A summary of the Sox2 staining is depicted below each staining. See also supplementary material Movie 1A,B. (E,F) Whole-mount immunostainings of E11.5 WT (E) and KO (F) embryos, showing a normal FoxA2 (blue) staining in the WT and KO, and a decrease or absence of keratin 8 (green) and Sox2 (red) expression in the KO cloaca compared with the WT. See also supplementary material Movie 2A,B. (G–J) Immunofluorescence analysis for the indicated markers of E11.5 WT and KO embryos. (G,H) WT cloaca epithelium (G) expresses keratin 19 (green) and Sox2 (red) at the distal region of the cloaca, in contrast to the KO, which shows no expression of keratin 19 or Sox2 (H). (I,J) CDX2 (red) is expressed at the proximal region of the cloaca epithelium in WT (I) and KO (J). All of the stainings have been performed on at least three WT and three KO littermates. A representative example for each antibody combination is shown. (K,L) H&E-stained sections of E11.5 WT (K) and *Shh* knockout (KO) (L) littermate embryos. (K) WT epithelium mostly consists of columnar epithelium, which, at two places, gradually transitions into pseudostratified columnar epithelium at two opposite ends. (L) The cloaca epithelium of *Shh* KO at E11.5 is predominantly pseudostratified columnar epithelium. Higher magnifications of the epithelium are shown in the inset. Scale bars: 100 μm (A), 50 μm (B–D), 5.000e4 μm (E,F), 20 μm (G–J), 10 μm (K,L), 5 μm (inset in K,L). The asterisk denotes autofluorescence. The dotted lines mark the epithelia. Arrows in E point to the nuclear expression of Sox2 and in I,J point to the nuclear expression of CDX2. Abbreviations: int, intestine; ec, ectoderm; cl, cloaca; hg, hindgut; ur, urethra; ugs, urogenital sinus.

Shh deficiency in mice results in cloaca malformation (Mo et al., 2001; Perriton et al., 2002; Seifert et al., 2009), although it is not known when defects in cloacal development are first apparent. Because little is known about cloacal development prior to septation, we generated a map of markers expressed during cloacal development from E10.5 through E13.5 (Fig. 1A–D). Because of the complex morphogenesis that accompanies the formation of cloacal derivatives, we analyzed the expression of markers using whole-mount immunofluorescence to generate three-dimensional images of the developing embryonic cloaca. We found that the cloaca at E10.5 expresses the simple-epithelium-type keratin 8 throughout the epithelium, whereas a population of cells positive for the transcription factor Sox2 is restricted to the dorsal side (Fig. 1A; supplementary material Fig. S1A; whole-mount supplementary material Movie 1A). Sox2 becomes more broadly expressed in the cloaca at E11.5 (Fig. 1B; supplementary material Fig. S1B) and will mark the future urethra and anal canal at later stages (Fig. 1C,D; supplementary material Fig. S1C,D; whole-mount supplementary material Movie 1B). Next, we analyzed molecularly and histologically this region in mouse at E11.5, before septation occurs and when the embryonic cloaca is still a cavity, to determine whether loss of Shh can affect differentiation of the cloaca epithelium. At that stage, Shh is strongly expressed in the wild-type (WT) cloaca epithelium (supplementary material Fig. S1E), in contrast to the knockout (supplementary material Fig. S1F). Molecularly, the WT embryonic cloaca epithelium was already compartmentalized, with the expression of Sox2 at the distal region (Fig. 1E,G) and the expression of CDX2 at the proximal region (Fig. 1I). We show that, in the *Shh* knockout epithelium, Sox2 is absent in the embryonic cloaca (Fig. 1F,H; whole-mount supplementary material Movie 2A,B), whereas CDX2 expression is unaffected (Fig. 1J). Moreover, the knockout embryonic cloaca epithelium shows an additional defect in keratin expression: keratin 8 is not uniformly expressed in these mutants, whereas it is in the WT (Fig. 1E compared with 1F; supplementary material Fig. S1G compared with S1H; whole-mount supplementary material Movie 2B), and keratin 19 is absent from the mutant epithelium (Fig. 1G compared with 1H). Histologically, whereas the WT cloaca epithelium is mostly comprised of columnar epithelium that, at two places, gradually transitions into pseudostratified columnar epithelium at two opposite ends (Fig. 1K), the *Shh* knockout cloaca shows predominantly pseudostratified columnar epithelium (Fig. 1L). These results indicate that Shh deficiency affects the molecular identity of the embryonic cloaca epithelium prior to septation.

### Similar features of cloaca malformations in mouse and humans, including a common channel with an indeterminate epithelial morphology

**Fig. 2.**
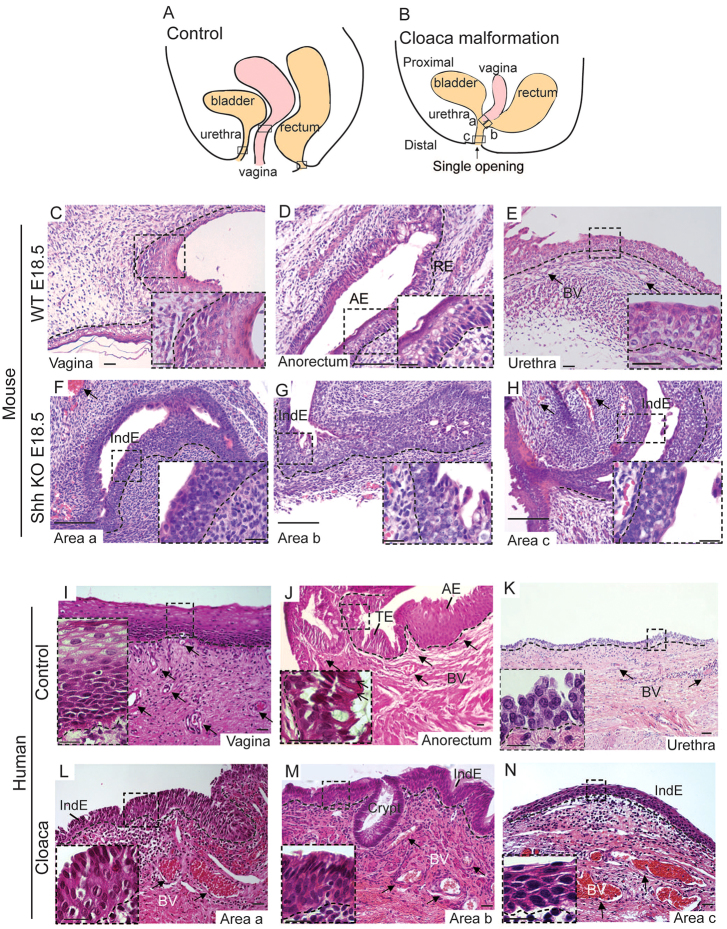
**The common channel in cloaca malformation of *Shh* deficient mice and in humans is composed of an indeterminate epithelium.**(A) Representation of normal cloaca development in a wild-type mouse and human control sample in which the urethra, vagina and rectum separate properly into three openings. The squares represent the area in which control tissues are obtained. (B) In cloaca malformation, the urethra, vagina and rectum fail to separate, and drain via a single common channel. Three areas shown by the squares and labeled a, b and c have been analyzed. Area a is closest to the vagina, area b is closest to the rectum and area c represents the most distal part of the common channel. (C–H) Hematoxylin and eosin (H&E)-stained mouse sections of E18.5 WT and *Shh* knockout (KO) littermate embryos. (C) Normal vaginal epithelium in WT embryo. Inset shows higher magnification of the stratified squamous epithelium with keratinization. (D) Normal anorectal region in WT embryo. Inset shows higher magnification of the anal region composed of stratified squamous epithelium with keratinization. (E) Normal epithelium in the urethra of a WT embryo magnified in the inset. (F–H) Area a, b and c of an *Shh* KO E18.5 embryo show stratified cuboidal epithelium, magnified in the inset. Arrows in E,F,H show blood vessels in the stroma. (I–K) H&E-stained human sections of control tissues including vagina (I), anorectum (J) and urethra (K). Insets show the stratified squamous epithelium of the vagina (I), the presence of goblet cells in the transitional epithelium (highlighted by the black arrows in the inset; J), and the pseudostratified columnar epithelium in the proximal part of the urethra (K). Black arrows show normal blood vessels in all control samples. (L,M) H&E-stained human sections of cloaca malformation specimen from area a (L), b (M) and c (N), showing indeterminate epithelium. Cloaca malformation specimen from area b shows a crypt in the middle of the indeterminate epithelium. In contrast to the tissue controls, the indeterminate epithelium does not contain goblet cells, squamous cells or umbrella cells. Black arrows show hypervascularity in the stroma surrounding the cloaca epithelium from all the areas analyzed (L,M). The dotted lines mark the epithelia. Scale bars: 20 μm (C–E,I,J,L-N and insets in C–K,M), 50 μm (F–H), 10 μm (K), 2 μm (insets of L and N). Abbreviations: TE, transitional epithelium; AE, anal epithelium; BV, blood vessel; IndE, indeterminate epithelium; RE, rectal epithelium; WT, wild type; KO, *Shh* knockout.

During normal development, the urethra, vagina and rectum separate into three distinct openings (Fig. 2A). In contrast, in *Shh* knockout mice and in patients with cloaca malformation, there is an improper septation of the urethra, vagina and rectum (Fig. 2B). We analyzed histologically and molecularly the epithelium of the common channel of *Shh* knockout at embryonic stage E18.5 from an area close to the vagina (Fig. 2B, area a) or the rectum (Fig. 2B, area b), or directly from the distal part of the common channel (Fig. 2B, area c, close to the single opening). At this late embryonic stage, septation is complete in WT embryos, with the formation of the vagina (Fig. 2C), anal canal/rectum (Fig. 2D) and urethra (Fig. 2E). We showed that all the areas analyzed (Fig. 2B, a, b and c) in *Shh* knockout mice are composed of stratified cuboidal epithelial cells that have a high nuclear:cytoplasmic (area) ratio; these cells are frequently lined on the luminal side by a single layer of squamous epithelium (Fig. 2F–H). These features are not consistent with either anal canal, vaginal canal (stratified squamous epithelium with keratinization) or urethra and potentially could be called indeterminate epithelium.

To determine whether these epithelial defects found in the *Shh* knockout mouse model can be translated into the human disease, we histologically analyzed 14 female patients from ages 6 to 48 months, each with a preoperative diagnosis of cloaca malformation. These patients underwent a posterior sagittal anorectovagino-urethroplasty to repair the defect and separate the common channel into three single openings (Levitt and Peña, 2010). Depending on the complexity of the cloaca malformation, samples were obtained from an area close to the vagina (Fig. 2B, area a) or the rectum (Fig. 2B, area b), or directly from the common channel (Fig. 2B, area c). Initial interpretations of the type of epithelium analyzed by three independent pathologists were based on hematoxylin and eosin (H&E) staining (Fig. 2I–N). Vagina control samples were obtained from the common wall of the fused duplex vaginas in three female patients (4-, 10- and 11-months old) (Fig. 2I). Anorectal control samples were obtained from the dentate line (anorectal transition zone) of one female (18-months old) and one male (12-years old) with colonic inertia who underwent a rectosigmoid resection (Fig. 2J), and urethra control sample was obtained from a 38-week-old female fetus (Fig. 2K). At this age the urethra epithelium is mature and comparable to a postnatal epithelium (Valdés-Dapenas, 1979). We show that the epithelium from cloaca patients obtained from area a (Fig. 2B) is composed of a mixture of morphologically normal vaginal-like, urothelial-like, colonic-like (summarized in Table 1) and a stratified columnar epithelium with no goblet cells (Fig. 2L). We define this epithelium as indeterminate because it cannot be classified pathologically and molecularly as a normal epithelium such as the stratified squamous shown in Fig. 2I, the transitional epithelium shown in Fig. 2J, or the pseudostratified columnar shown in Fig. 2K. Area b (Fig. 2B) contained morphologically normal-looking colonic-like, urothelial-like and transitional-like epithelium, and indeterminate epithelium (Fig. 2B,M). Area c (Fig. 2B) only contained epithelium composed of basal- and intermediate-like cells, but without umbrella cells on the top layer (Fig. 2N) as are normally found in bladder (Stacey, 2012). Therefore, we refer to this epithelium as also being indeterminate. In one case from area c (case 12, Table 1), we found crypts, like those found in area b (Fig. 2M), to be intercalated in the indeterminate epithelium.

**Table 1.**
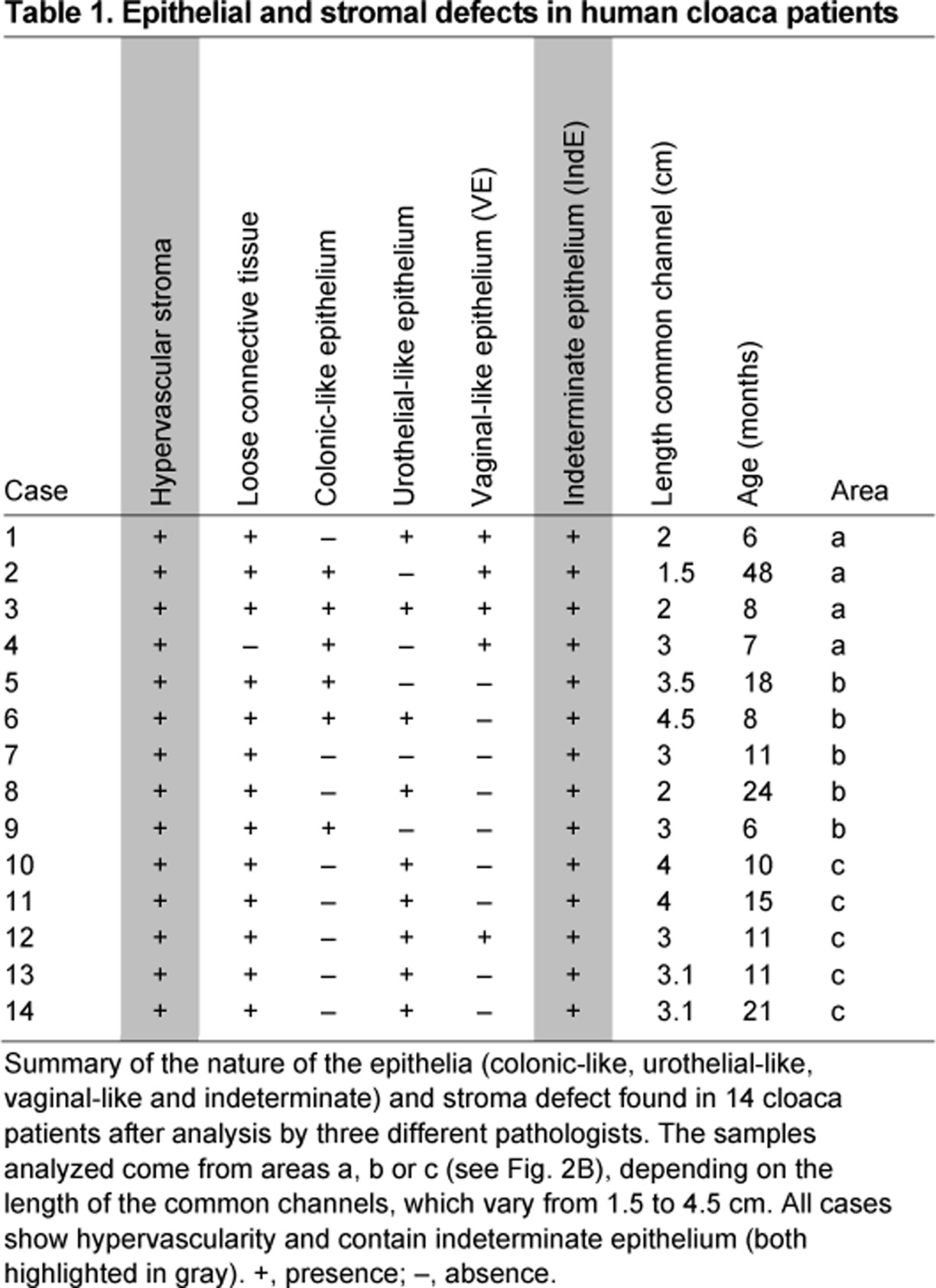
Epithelial and stromal defects in human cloaca patients

In summary, indeterminate epithelium was found in all specimens within or directly surrounding the common channel in humans, and this is paralleled in the *Shh* knockout mouse.

### Shh is decreased in the epithelium of cloaca patients

**Fig. 3.**
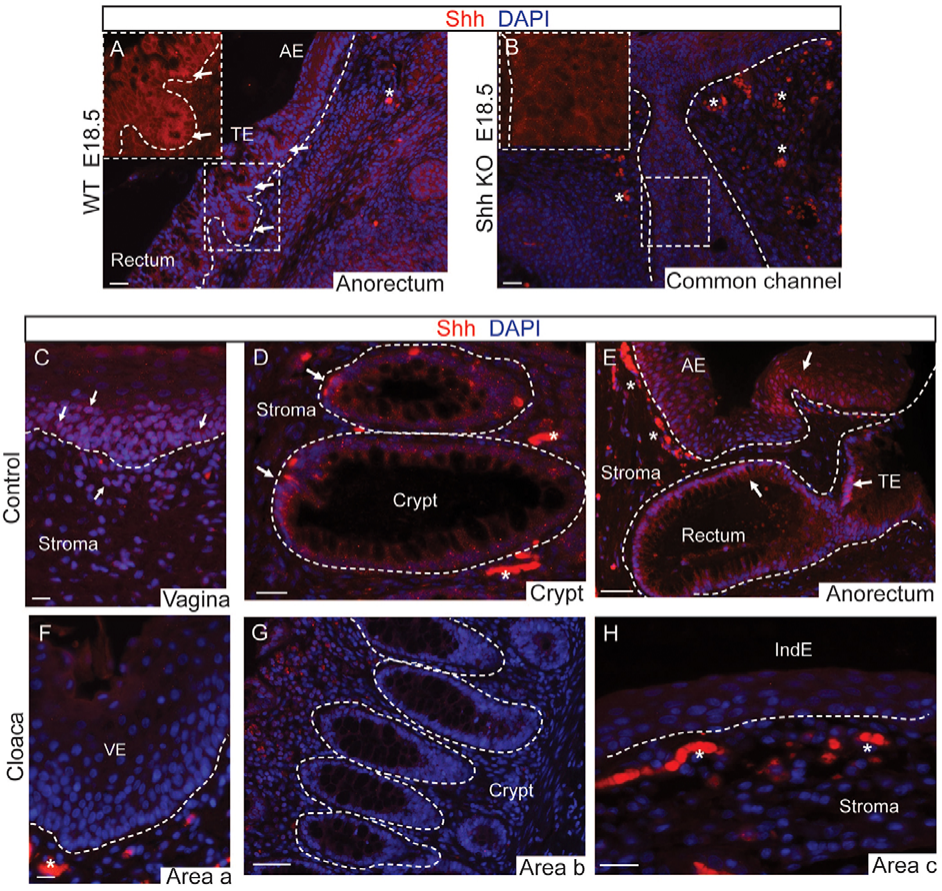
**Shh deficiency in epithelium of human cloaca patients.** (A,B) In mouse, Shh (red) labels the anal epithelium in E18.5 WT embryos (A), whereas it is absent in the *Shh* knockout (KO) (B). (C–H) Immunofluorescence analysis of Shh (red) in epithelium and stroma of human control tissues from vagina (C), crypt region (D) and the anorectum (E), and common channel from area a (F), area b (G) and area c (H) (areas are shown in Fig. 2B). The white arrows show expression of Shh in epithelia and stroma in control tissues. See supplementary material Table S1 for details of all cases analyzed. The dotted lines mark the epithelia. Scale bars: 50 μm (E,G), 20 μm (AD,F,H). Abbreviations: AE, anal epithelium; TE, transitional epithelium; VE, vaginal-like epithelium; IndE, indeterminate epithelium. The asterisks denote autofluorescence.

Shh is known to play a central role in cloacal morphogenesis. For example, cloacal septation depends on paracrine signaling by Shh from the cloacal endoderm to the mesenchyme (Haraguchi et al., 2001; Mo et al., 2001; Perriton et al., 2002; Haraguchi et al., 2007; Lin et al., 2009; Seifert et al., 2009; Seifert et al., 2010). We therefore investigated whether Shh expression might be decreased in the tissue from cloaca patients. The specificity of the Shh immunostaining was confirmed using *Shh* knockout tissues (Fig. 3A,B). In humans, Shh was expressed in control tissues (vagina, Fig. 3C; colorectal epithelium, 3D; and the transition epithelium, which is the zone between the anorectal mucosa in idiopathic prolapse, 3E), but was reduced or absent in 86% of cloaca samples (*n*=12/14 tested) (Fig. 3F–H, summarized in supplementary material Table S1). The reduction in Shh expression in the cloaca malformation samples suggests that perturbation of Shh signaling might be a common feature in individuals with cloacal malformations.

### Molecular defects in the common channel of cloaca malformations in mice and humans

**Fig. 4.**
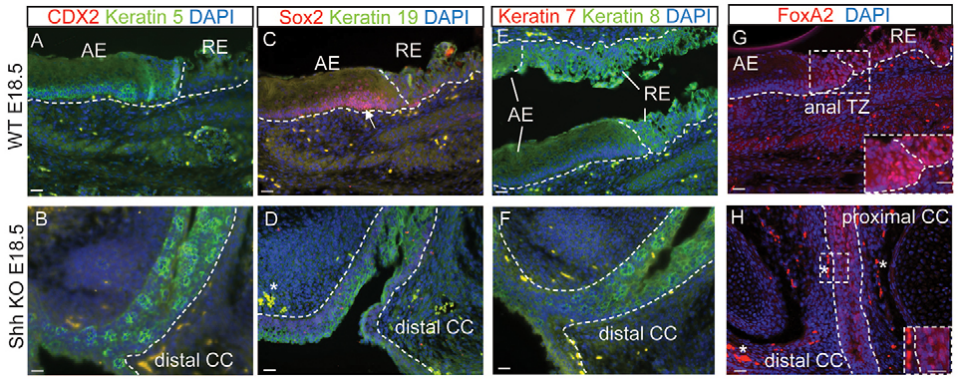
**The common channel of *Shh* mutant mice shows differentiation defects resulting in an indeterminate epithelium.** (A–H) Immunofluorescence analysis of WT and *Shh* knockout (KO) E18.5 embryo for the indicated markers. (A,B) Keratin 5 (green) marks the anal epithelium in WT (A) and the distal common channel in KO (B). CDX2 (red) is absent in the distal common channel (B). (C,D) Sox2 (red) marks the anal epithelium in WT as denoted by the white arrow (C) and is absent in the distal common channel (D). Keratin 19 (green) marks the WT rectum (C) and the suprabasal layer of the distal common channel (D). (E,F) Keratin 8 (green) labels the rectum epithelium in WT embryo (E) and the distal common channel in KO (F), whereas keratin 7 (red), normally found in urothelium, is absent in the distal common channel. (G,H) FoxA2 (red) marks the anal transition zone and the rectal epithelium in the E18.5 WT embryo (G) and the proximal common channel in the KO, but is absent in the distal region (H). All of the stainings have been performed on at least three WT and three KO littermates. A representative example for each antibody combination is shown. The dotted lines mark the epithelia. Abbreviations: RE, rectal epithelium; AE, anal epithelium; CC, common channel; TZ, transition zone. All scale bars: 20 μm. The asterisk denotes autofluorescence.

To help further determine the origin of the indeterminate epithelium in mice and humans, we used markers of each type of epithelium: stratified squamous (anal and vaginal epithelium), simple (rectum) and transitional. In mouse, keratin 5 is found in normal stratified squamous epithelium with keratinization (anal canal) (Fig. 4A) and the simple-epithelium-type cytokeratins keratin 8 and 19 are found in normal rectum (Fig. 4C,E). Interestingly, both the stratified-epithelium-type keratin 5 and the simple-epithelium-type keratin 8 and 19 are expressed in the common channel of *Shh* knockout mice (Fig. 4B,D,F). Keratin 7, normally present in urethra, is not expressed in the common channel in these knockouts (Fig. 4F). Moreover, Sox2 is normally present in the anal canal of WT mice (Fig. 4C) but is absent from the common channel in the knockouts (Fig. 4D). Finally, the distal common channel did not express the endodermal marker FoxA2, normally present in the anal transition zone (Fig. 4G), whereas it was expressed in the proximal part in the knockouts (Fig. 4H), which suggests that this epithelium has undergone maturation and specification. These data indicate that the common channel in *Shh* deficient mice does not share the molecular characteristics of a normal anal canal, rectum, vagina or urethra.

**Fig. 5.**
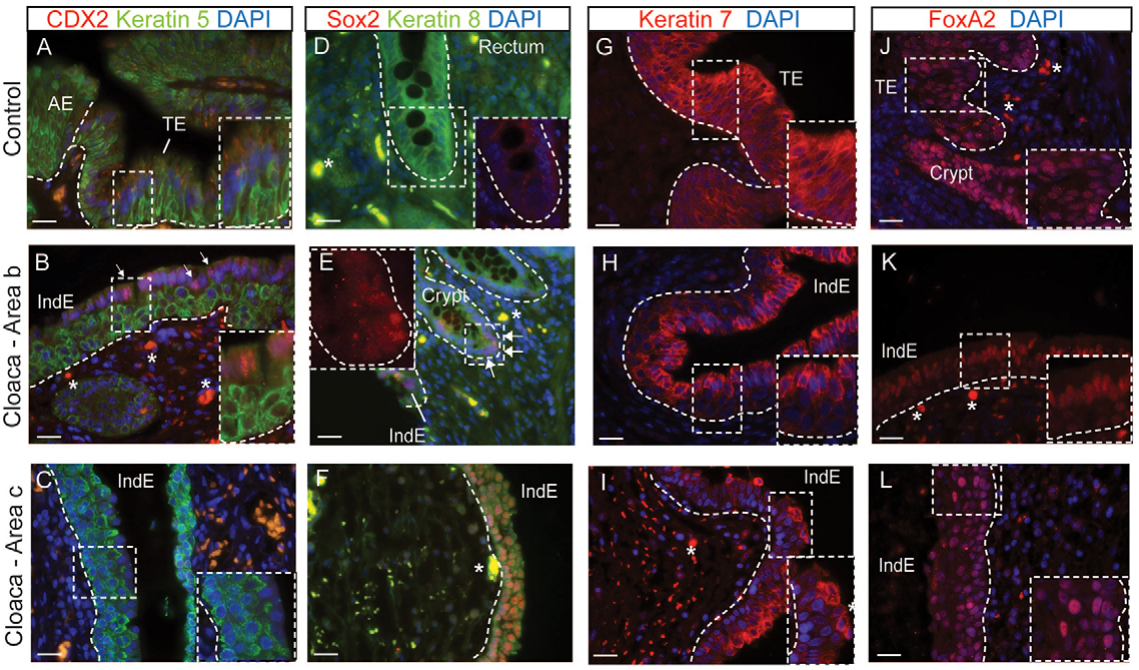
**Molecular identity of the indeterminate epithelium in human cloaca patients.** (A–L) Immunofluorescence analysis for the indicated markers in control tissue (A,D,G,J), cloaca from area b (B,E,H,K) and cloaca from area c (C,F,I,L). (A–C) The indeterminate epithelium in area b (B) is molecularly distinct from the normal transitional epithelium (A) because it expresses CDX2 (red) in the suprabasal layer of cells (as denoted by the white arrows in B and magnified in the inset) whereas basal cells express keratin 5 (green). (C) The indeterminate epithelium in area c is also distinct from the control tissue, because the layer of keratin-5-positive cells is increased. (D–F) Sox2 (red), not normally found in crypt from the control tissue (D), is abnormally expressed in a crypt found in the indeterminate epithelium of a cloaca sample from area b as denoted by the white arrows (E). Keratin 8 (green), marking simple epithelia of the crypt (D), is not expressed in the indeterminate epithelium present in cloaca from area c (F). (G–I) The indeterminate epithelium in area b and c is distinct from the control tissue as denoted by the expression of keratin 7 (red) throughout the transitional epithelia in controls (G), in contrast to the restricted expression in the suprabasal layer of cells in cloaca areas b and c (H,I). The indeterminate epithelium in cloaca from area b is more specified than the cloaca from the area c, as denoted by the expression of keratin 7 uniformly throughout the suprabasal layer of cells (H), in contrast to area c, in which keratin 7 is present in some suprabasal cells of the indeterminate epithelium (I). (J–L) The indeterminate epithelium in cloaca patients expresses endodermal markers. (J) FoxA2 (red) is expressed in the crypt and transitional epithelium in control tissue. (K) FoxA2 is mostly expressed in the suprabasal layer of the indeterminate epithelium of the cloaca from area b. (L) In area c, FoxA2 is present uniformly in the epithelium. The dotted lines mark the epithelia. Abbreviations: AE, anal epithelium; TE, transitional epithelium; IndE, indeterminate epithelium. The asterisk denotes autofluorescence. All scale bars: 20 μm.

Human control anal epithelium was positive for keratin 5, p63 and Sox2, and negative for keratin 8, keratin 7, CDX2 and FoxA2 (Fig. 5A; supplementary material Fig. S2A–D); control rectum epithelium was negative for keratin 5, p63, Sox2 and keratin 7, and positive for keratin 8 and CDX2 (Fig. 5D; supplementary material Fig. S2E–H); control transitional epithelium was positive for keratin 5, p63, Sox2, keratin 8 and keratin 7, and negative for CDX2 (Fig. 5A,G; supplementary material Fig. S2A,B). We found that the indeterminate epithelium from areas a and b (Fig. 2B) are molecularly very similar (supplementary material Fig. S2) and express markers that are absent in control transitional epithelium and control rectum. For example, CDX2, which is normally expressed in developing mouse hindgut endoderm (van de Ven et al., 2011) and rectum (supplementary material Fig. S2H), is absent from the transitional epithelium (Fig. 5A) and is present in the suprabasal layer of cells in the indeterminate epithelium in cloaca from the area b (Fig. 5B). In addition, we determined that crypts found in the indeterminate epithelium express keratin 8 of simple epithelia and the transcription factor Sox2, not normally found in normal rectum (Fig. 5D,E). Those results complement the pathological analysis by showing that indeterminate epithelium at the molecular level shows alterations. The indeterminate epithelium from region c (Fig. 2B) is also unique and does not resemble any control epithelium (Fig. 5). It expresses all markers found in transitional epithelium, such as keratin 5 (Fig. 5C), p63 (supplementary material Fig. S2N), FoxA2 (Fig. 5L) and Sox2, with the exception of keratin 8 (Fig. 5F).

By comparing the expression of those molecular markers between the indeterminate epithelium from the more proximal region (such as area a and b) and the distal region (area c), we found that the epithelium seems to be more specified, correlating with the location close to the vagina and rectum (Fig. 2B). This is illustrated with the expression of keratin 8 in the suprabasal layer of the indeterminate epithelium from area a and b (supplementary material Fig. S2M,K compared with S2N) and the uniform expression of keratin 7 throughout the suprabasal layer of cells in area b and not in area c (Fig. 5H compared with 5I). Additionally, the expression of the endodermal marker FoxA2 is found throughout the distal common channel in cloaca from area c (Fig. 5L), in contrast to the top layer of the indeterminate epithelium in cloaca from area a and b (Fig. 5K; supplementary material Fig. S2L). Altogether, these results suggest that, histologically and at the molecular level, the cells found in the common channel of the *Shh* mutant mouse model and in human cloaca patients might have undergone abnormal differentiation, resulting in an epithelium distinct from any known adult epithelium.

### Hypervascularity in the stroma surrounding the common channel of mouse and human cloaca malformation

**Fig. 6.**
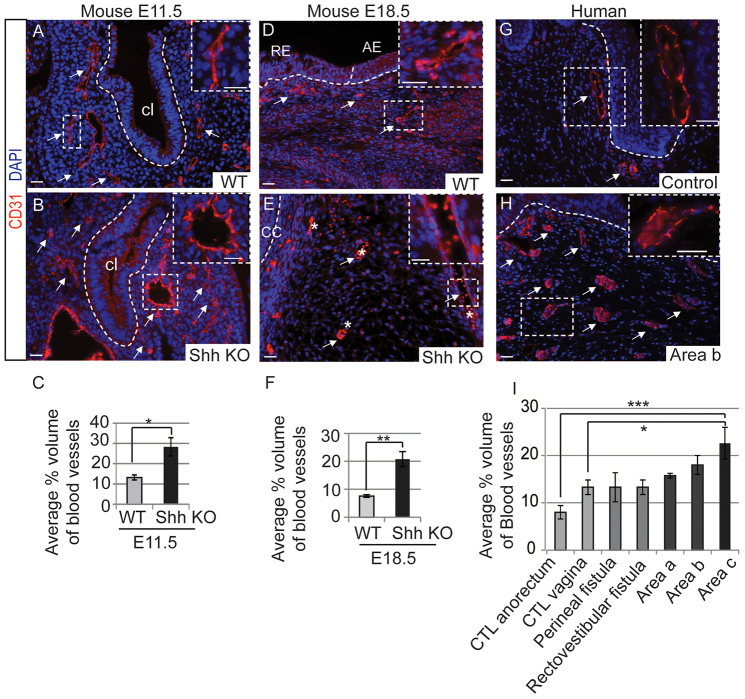
**Hypervascularity in the stroma surrounding the common channel in mice and humans with cloaca** malformation. (A,B) Immunofluorescence staining of endothelial cells surrounding blood vessels with CD31 (red) in the stroma of WT (A) and *Shh* knockout (KO) (B) mice at E11.5. Note that the vessels are enlarged in the KO but not malformed because CD31 is normally expressed. (C) There was a significantly higher percentage volume of blood vessels in *Shh* KO stroma at E11.5 stages compared with WT littermates. Quantification and histological interpretation have been performed on three WT and three KO littermates at each embryonic stage. *P=0.005. (D,E) Immunofluorescence staining of endothelial cells surrounding blood vessels with CD31 (red) in the stroma of WT E18.5 anorectal region (D) and a KO littermate (E). Note that the vessels are enlarged in the KO but not malformed because CD31 is normally expressed (see higher magnification in the inset). The asterisk denotes autofluorescence of the red blood cells. (F) There was a significantly higher percentage volume of blood vessels in *Shh* KO stroma at E18.5 stage compared with WT littermates. Quantification and histological interpretation have been performed on three WT and three KO littermates. **P=0.001. (G,H) Immunofluorescence staining of endothelial cells surrounding blood vessels with CD31 (in red) in the stroma of normal vagina (G) and cloaca from area b (H). Higher magnification of a blood vessel, showing CD31 expression is shown in the inset. (I) Vascularity has been quantified by comparing the average of the percentage volume of blood vessels between control anorectum (*n*=3), control vagina (*n*=3), perineal fistula (*n*=6), rectovestibular fistula (*n*=3), cloaca from area a (*n*=4), cloaca from area b (*n*=5) and cloaca from area c (*n*=5). *P<0.05, ***P<0.001. The dotted lines mark the epithelia. The white arrows show blood vessels. All scale bars: 20 μm. Abbreviations: cl, cloaca; RE, rectal epithelium; AE, anal epithelium; CC: common channel; CTL, control.

We next analyzed the stroma surrounding the cloaca epithelium of *Shh* knockout mice at E11.5 (Fig. 6A–C) and E18.5 (Fig. 6D–F), and we found a significant increase in the number of blood vessels when compared with control tissues at both ages (quantified in Fig. 6C,F). The blood vessels in *Shh* knockout stroma are dilated but not malformed: the wall of the vessels look pathologically normal (Fig. 1L; Fig. 2H) and the expression of the endothelial marker CD31 is similar to control tissues (Fig. 6A,B,D,E). In humans, the urogenital system and anorectum region is normally highly vascularized compared with other parts of the body (Masumoto et al., 2011). When compared with the normally highly vascularized stroma of our controls (Fig. 2I–K), the stroma of individuals with cloaca malformation show an even higher degree of hypervascularity (Fig. 2L–N). Moreover, there is a significant increase in the number of blood vessels in the stroma of cloaca malformation samples coming from the distal common channel (region c) when compared with control tissues (quantified in Fig. 6I). Consistent with our finding in mouse, the vessels are dilated but not malformed: the vessel walls appear pathologically normal (Fig. 2L–N) and the expression of the endothelial marker CD31 is similar to control tissues (Fig. 6G–H). No chronic inflammation in the stroma surrounding the cloaca malformation samples has been detected. In order to see whether the increased hypervascularity seen in the stroma was specific to cloaca, we histologically analyzed other anorectal malformations, such as perineal fistula and rectovestibular fistula, which are more benign with excellent functional prognosis (Levitt and Peña, 2007). We determined that the stroma of perineal fistula and rectovestibular fistula are not as highly vascularized as in cloaca patients (quantification in Fig. 6I). These results suggest that the hypervascularity defects are also seen as a spectrum. Moreover, the difference in the mesenchyme persists in human patients past early development, suggesting that those defects occur embryonically. Altogether, the abnormal surrounding stroma suggests that a differentiation defect might have occurred during development, resulting in the formation of epithelia expressing aberrant transcription factors.

### BMP signaling is decreased in the cloaca epithelium and stroma in mice and humans with cloaca malformation

**Fig. 7.**
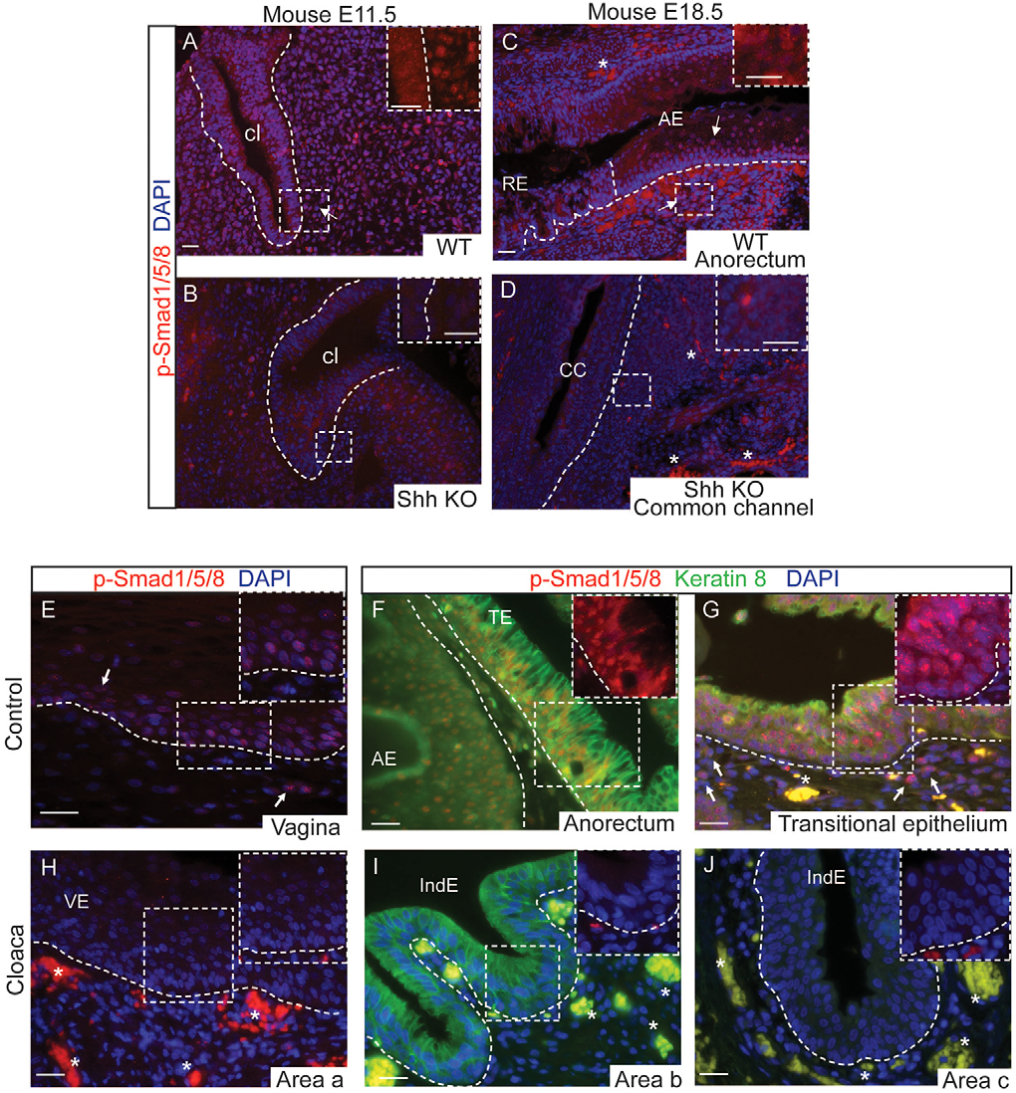
**BMP signaling deficiency in epithelium and stroma of *Shh* deficient mice and in human cloaca patients.** (A–J) Immunofluorescence analysis of p-Smad1/5/8 (red) alone or in combination with keratin 8 (green) to label simple epithelia. (A–D) BMP signaling deficiency [stained with p-Smad1/5/8 antibody (red)] in epithelium and stroma of a *Shh* KO embryo at E11.5 compared with a WT littermate (A,B) and at E18.5 (C,D). Three WT and three KO littermates have been analyzed. (E–G) Control tissues show nuclear expression of p-Smad1/5/8 in vaginal (E), anal and transitional epithelia (F,G) and in the stroma (E,G; white arrows). (H–J) p-Smad1/5/8 is predominantly absent in the epithelium and stroma of all cloaca malformation samples analyzed (*n*=11 negative expression in 14 samples analyzed) (see supplementary material Table S1). Area a shows vaginal-like epithelium (H), and areas b and c show indeterminate epithelium (I,J). The dotted lines mark the epithelia. All scale bars: 20 μm. Abbreviations: cl, cloaca; AE, anal epithelium; TE, transitional epithelium; RE, rectal epithelium; IndE, indeterminate epithelium; VE, vaginal-like epithelium; CC, common channel. The asterisk denotes autofluorescence.

Given our findings of hypervascularity and Shh deficiency in the stroma and epithelium of mouse and human cloaca malformation samples, we wondered whether BMP signaling, known to control blood-vessel formation in zebrafish (Pyati et al., 2006) and involved in cloacal septation (Stickney et al., 2007; Wu et al., 2009), might also be dysregulated in cloaca malformation. We performed immunofluorescence stainings on sections for the phosphorylated forms of Smad1/5/8 (p-Smad1/5/8) as a readout of BMP signaling (Fig. 7). Whereas nuclear p-Smad1/5/8 expression was detected in the stroma of WT mouse embryos at E11.5 (Fig. 7A), it was absent in the stroma of *Shh* knockouts (Fig. 7B). This defect was still observed at later stages at which p-Smad1/5/8 is normally present in the epithelium and stroma of the WT anorectal region (Fig. 7C) but was absent in the *Shh* knockout common channel and surrounding stroma (Fig. 7D). Similarly, whereas nuclear p-Smad1/5/8 expression was detected in the epithelium and stroma of human control tissues (Fig. 7E–G), it was absent in the epithelium and stroma of 78.6% cloaca samples (*n*=11/14 tested) (Fig. 7H–J; summarized in supplementary material Table S1). In some cases, we found p-Smad1/5/8 expression in the indeterminate epithelium of cloaca malformation samples but the expression was always negative in the stroma (supplementary material Table S1, case 11).

Taken together, these findings suggest that the loss of Shh contributes to the dysregulation of BMP signaling in the stroma. BMP signaling is important for proper control of blood-vessel formation, and the observed dysregulation could contribute to the abnormal epithelial differentiation and might account for the failed septation in cloaca malformation. Moreover, the hypervascularity and BMP signaling defects are similarly found in the stroma of *Shh* knockout mice before septation, suggesting that these defects occur early during development.

## DISCUSSION

Despite the clinical impact of cloaca malformations, this congenital disease is understudied (Wong et al., 2013) and the cellular nature of the epithelium found in the common channel resulting in the cloaca malformation has been poorly analyzed. Our analyses of 14 human cloaca malformation specimens clearly show that the human common channel contains an indeterminate epithelium with molecular alterations, displays an increased hypervascular stroma and has reduced or absent Shh expression in the epithelium. Similarly, we found that the stroma surrounding the cloaca malformation in *Shh* mutant mice is also hypervascular when analyzed at two embryonic stages: prior to septation (E11.5) and after septation (E18.5). Although our mouse study using E11.5 *Shh* mutant embryos shows an early differentiation defect, such results in human (E11.5 mice correspond to a 10-week-old fetus) are only speculative because such samples are rare and difficult to obtain. Our results using human cloaca malformation samples from 6- to 48-month-old individuals suggest that a differentiation defect might have occurred during development and that human cloaca malformations can result from endoderm mis-patterning, resulting in abnormal differentiation prior to septation into hindgut and urogenital systems.

### Perturbed BMP signaling in cloaca malformation

Furthermore, we also identified a reduction or loss of p-Smad1/5/8 activity in *Shh* knockout mice as well as human cloaca malformation specimens, suggesting that BMP signaling is negatively affected. Based on our data, as well as on studies in mouse and zebrafish (Pyati et al., 2006; Parkin et al., 2009; Wu et al., 2009; Xu et al., 2012), we speculate that disruption of epithelial hedgehog signaling results in alterations in BMP signaling in the surrounding mesenchyme. This causes early abnormal differentiation of cloacal endoderm as well as subsequent hypervascularity in the stroma surrounding the cloaca. Our data are in accordance with previous work showing that the pericloacal mesenchyme surrounding the embryonic cloaca expresses BMP4 (Perriton et al., 2002) and that mouse embryos with induced anorectal malformation show an absence of Shh and BMP4 (Sasaki et al., 2004). This signaling relay between Shh signaling and BMP has also been shown to be essential for mouse urinary tract morphogenesis (Haraguchi et al., 2012).

### Insights into altered epithelial development in cloaca malformation

The term cloaca can be used to describe a transient embryonic structure in human embryos, a congenital anomaly, and a normal organ in birds, amphibians and reptiles. This can lead to the false suggestion that the cloaca has the same characteristics in those three conditions. The nature of the epithelium found in the normal human cloaca region in embryos is described as a columnar epithelium that is most likely pseudostratified (van der Putte et al., 2008). In birds, amphibians and reptiles, the cloaca is a short canal where the genital and urinary ducts, the rectum, and the bladder converge. In reptiles and birds, the cloaca is subdivided into three regions called the coprodeum, urodeum and proctodeum, which are lined by different epithelium according to the organ that merges into it. The coprodeum and urodeum reflect the hindgut type of epithelium and the proctodeum is lined by stratified squamous epithelium. Therefore, it does not represent a common channel as in human cloaca malformations, but more a simple opening.

Our study demonstrates that the epithelium from the common channel of cloaca patients is indeterminate, suggesting that cells might have undergone abnormal differentiation resulting in an unclassified epithelium. Our data also suggest that the developmental program of the epithelium in cloaca patients might be altered, because a transcription factor, Sox2, normally found in stratified epithelium in adults, is now found in simple epithelia. Cloaca malformation is also defined as ‘persistent cloaca’ because it is thought that it might result from an arrest of the division of the embryonic cloaca. However, our results using the *Shh* knockout mice show that the epithelium from the common channel has matured and does not show the same characteristics as a cloaca epithelium before septation, suggesting that the epithelium from the cloaca malformation does not result in the persistence of the normal cloaca epithelium found in the early stage of development. Therefore, we favor a model of defective epithelial-mesenchymal cross-talk in which loss of Shh in the epithelia results in abnormal mesenchymal responses.

Despite a good surgical reconstruction in cloaca patients (Peña and Devries, 1982), ~25% of patients require some type of vaginal replacement with bowel during the main repair or later in life. The neovagina epithelium carries the possibility of a future malignant potential (Schober, 2007; Idrees et al., 2009). The new vaginal epithelium created with tissue from the rectum creates a transition zone where the tissue is suddenly subjected to new contacts or stresses. We and others have shown that those transition zones are susceptible to tumor formation in human and mouse (Guasch et al., 2007; Runck et al., 2010; McNairn and Guasch, 2011). Therefore, identifying the molecular basis for cloaca malformation would allow for prenatal diagnosis as well as provide a molecular foundation for tissue engineering efforts that could aid in better surgical reconstruction.

## MATERIALS AND METHODS

### Mice and genotyping

The B6.Cg-*Shh^tm1(EGFP/cre)Cjt^*/J mice in a C57BL/6 background were purchased from The Jackson Laboratory (Bar Harbor, Maine, USA). Knockout female embryos were obtained by mating heterozygous parents and genotyping was conducted by PCR of tail skin DNAs using mouse Cre and Y chromosome primers. All experiments were approved by the Cincinnati Children’s Hospital Research Foundation Institutional Animal Care and Use Committee and carried out using standard procedures.

### Patient samples

Fourteen female patients were included in the study based upon the clinical diagnosis of cloaca at the Colorectal Center for Children at Cincinnati Children’s Hospital Medical Center. Other anorectal malformation samples include perineal fistula (*n*=6) and rectovestibular fistula (*n*=3). Control specimens include vagina, anorectum and urethra. Vagina tissue was obtained from vaginal septum (*n*=3), and anal canal, transitional epithelium and rectum from idiopathic prolapse surgery (*n*=3). Urethral tissue was obtained from the biopsy of a 38-week-old female fetus. The various samples were collected as a surgical waste with information provided regarding the age, sex and length of the common channel (cm) of the donors with Institutional Review Board (IRB) approval at Cincinnati Children’s Hospital Medical Center. The IRB determined that the research does not meet the regulatory criteria for research involving human subjects because there were no interaction with the donors and no identifiable private information. Depending on the cases, the samples received came from an area distal to the vagina (a), the rectum (b) or directly from the most distal part of the common channel (c) (see Fig. 2B).

### Histological and immunofluorescent analysis

Samples from the human cloaca malformations, human control specimens, *Shh* knockout mice and WT mouse embryos were fixed in formalin, embedded in paraffin, and 5-μm-thick sections were cut and stained with H&E. The epithelium and the stroma were analyzed by at least three independent pathologists at Cincinnati Children’s Hospital Medical Center. Paraffin sections were melted in a 60°C oven overnight, followed by deparaffinization in xylene, and then rehydrated in a graded series of ethanol rinses. The tissues were treated with heat-assisted antigen retrieval in 0.01 M sodium citrate buffer (pH 6.0). Immunostainings were performed as previously described (Runck et al., 2010). Briefly, tissues were permeabilized using 0.1% Triton-PBS and non-specific staining was eliminated via the following blocking solution: 2.5% normal goat serum (Jackson ImmunoResearch Laboratories, West Grove, PA), 2.5% normal donkey serum (Jackson ImmunoResearch Laboratories, West Grove, PA), 2% gelatin (Sigma, St Louis, MO), 0.1% Triton X-100 (Sigma, St Louis, MO) and 1% BSA (Sigma, St Louis, MO) in 1× PBS. For stains using mouse monoclonal antibodies on mouse tissues, the MOM kit (Vector Laboratories, Burlingame, CA) was used.

Primary antibodies against the following proteins were used at the dilution indicated: keratin 5 (Seven Hills Bioreagents, Cincinnati, OH; 1:2000), keratin 8 and 19 (these antibodies, developed by Dr Brulet and Dr Kemler, were obtained from the NICHD Developmental Studies Hybridoma Bank maintained by the University of Iowa; 1:10), keratin 7 (Cell Signaling, Beverly, MA; 1:100), p63 (Santa-Cruz Biotechnology Inc., Santa Cruz, CA; 1:50), FoxA2 (Santa-Cruz Biotechnology Inc., Santa Cruz, CA; 1:250), Shh (Santa Cruz Biotechnology Inc., Santa Cruz, CA; 1:2000), Sox2 (Seven Hills Bioreagents, Cincinnati, OH; 1:5000), CDX2 (Biogenex, Fremont, CA; 1:250), p-Smad1/5/8 (Cell Signaling, Beverly, MA; 1:500), CD31 (Abcam, Cambridge, MA; 1:50). 4′,6-diamidino-2-phenylindole (DAPI) was used as a marker of cell nuclei (Sigma Chemical Co., St Louis, MO; 1:1000). Secondary antibodies Alexa-Fluor-488 and -555 (Invitrogen Corporation, Carlsbad, CA) were used at a dilution of 1:1000. Immunostained sections were analyzed using a fluorescent microscope AxioImager M1 (Zeiss) and pictures were taken with an axioCam MRm camera (Zeiss). Images in different focal planes were combined using the Extended Focus Module within the Axiovision software suite (Zeiss).

### Whole-mount immunostaining

E11.5 embryos were harvested and dissected with microsurgical instruments under a dissecting microscope to remove the limbs and the upper part of the embryos. After fixation in 4% paraformaldehyde overnight at 4°C, the embryos were washed briefly in 1× PBS, transferred to 100% methanol and placed in a solution of methanol, DMSO and H_2_O_2_ at a ratio of 4:1:1, respectively, for 2 hours. The embryos were then rehydrated in a graded series of methanol rinses (100%, 75%, 50% and 25%). Immunostainings were performed using a TSA blocking reagent (Invitrogen TSA kit T20922 component D) diluted in 0.5% Triton PBS1X (PerkinElmer, Hebron, KY) and the primary antibodies against the following proteins were used: keratin 5 (guinea pig, Guasch Lab; 1:100), CD31 (Abcam, Cambridge, MA; 1:500), FoxA2 (Santa-Cruz Biotechnology Inc., Santa Cruz, CA; 1:250). Secondary antibodies (donkey anti-goat Alexa Fluor 488, donkey anti-rabbit Cy3, donkey anti-guinea-pig Dylight 649; Jackson ImmunoResearch Laboratories, Inc., West Grove, PA) were used at a dilution of 1:500. After 3 hours of washes in 0.5% Triton 1× PBS, the stained embryos were rinsed in 100% methanol and placed in a solution containing benzyl alcohol and benzyl benzoate at a ratio of 1:2, respectively, 30 minutes prior to imaging. Immunostained embryos were imaged on a Nikon A1R-Si laser scanning confocal on a Nikon Eclipse Ti inverted microscope. Whole-mount movies and stacked images were made and analyzed using Imaris 7.6.4 software.

### Quantification

The volume percentage of blood vessel was quantified using an eyepiece net micrometer KR-406, with grid matrix of 10 mm×10 mm, with total of 100 grid squares, resulting in each grid square of dimension 1 mm×1 mm (Carl Zeiss Microscopy, LLC). There are 100 intersecting points on the grid. Using a 10× objective, the entire section of each tissue was scored. The number of points on blood vessels was counted on each field if the intersecting point fell on blood-vessel wall or within the lumen of the vessel. The number of points on blood vessels divided by the total number of points yields the volume percent (Chalkley et al., 1949).

### Statistics

Data are expressed as means ± s.d. Comparison between two groups was performed using unpaired two-tailed Student’s *t*-test. *P*<0.05 was considered significant.

## Supplementary Material

Supplementary Material
