# Supplementary material for: Defining the molecular pathologies in cloaca malformation: similarities between mouse and human
Source: Dis Model Mech. 2014 Feb 13;7(4):483–93. doi: 10.1242/dmm.014530 (PMC3974458; doi:10.1242/dmm.014530)
Supplement: Supplementary Material [file supp_7_4_483__index.html]

Defining the molecular pathologies in cloaca malformation: similarities between mouse and human — Supplementary Material 

# Defining the molecular pathologies in cloaca malformation: similarities between mouse and human

## DMM014530 Supplementary Material

**Files in this Data Supplement:**

- **Supplementary Material**
