# Supplementary material for: Defining the molecular pathologies in cloaca malformation: similarities between mouse and human
Source: Dis Model Mech. 2014 Feb 13;7(4):483–93. doi: 10.1242/dmm.014530 (PMC3974458; doi:10.1242/dmm.014530)
Supplement: Supplementary Material [file supp_7.4.483_DMM014530.pdf]

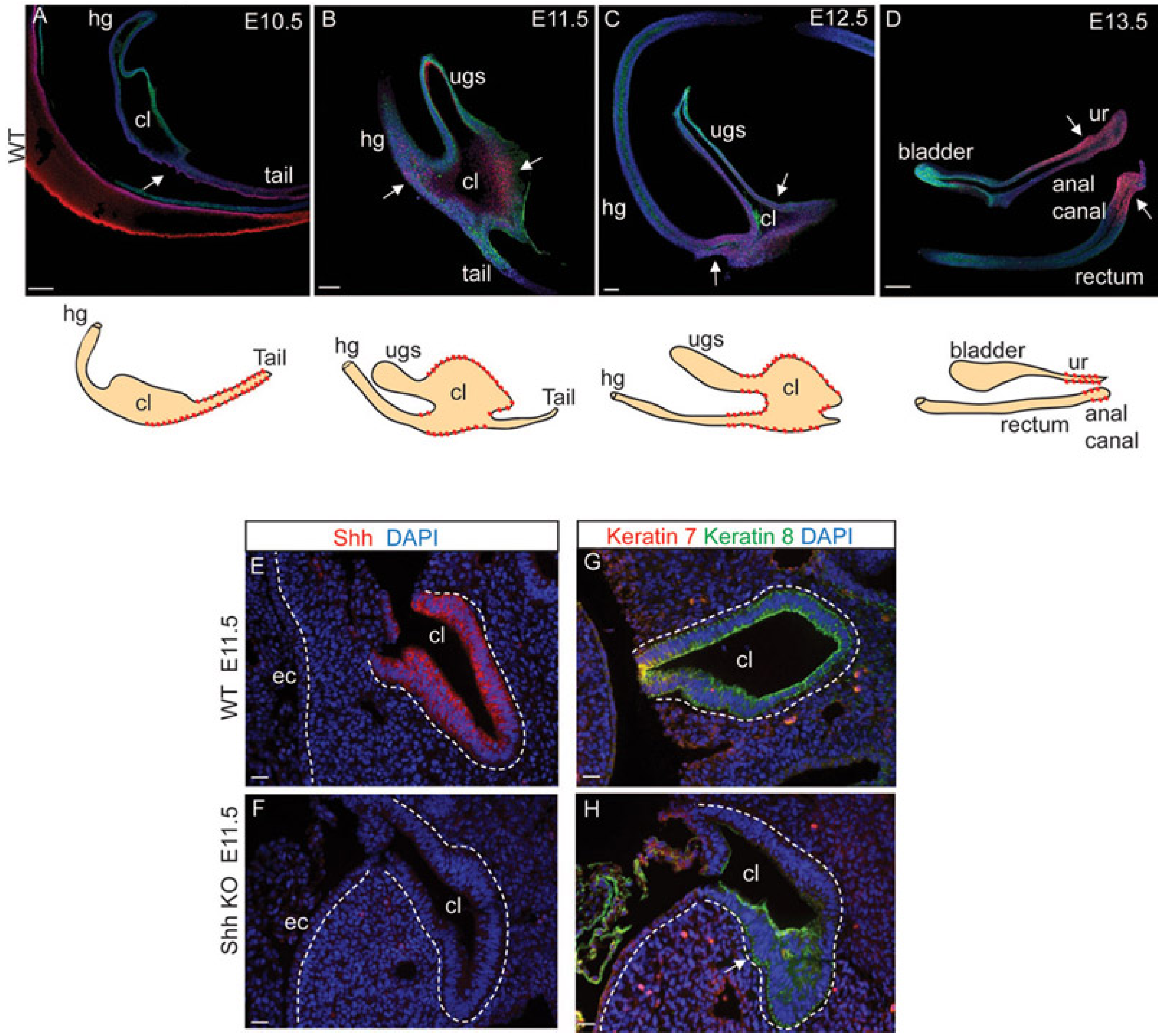

Fig. S1: Early patterning of the normal cloaca and molecular defects in the cloaca epithelium of *Shh* knockout embryos. (A-D) Slices from whole mount immunofluorescence (Fig.1) showing early patterning of the WT cloaca epithelium at E10.5 (A), E11.5 (B), E12.5 (C) and E13.5 (D) embryos stained with Sox2 (red), Keratin 8 (green) and FoxA2 (blue). A summary of the Sox2 staining is depicted below each staining. (E,F) Immunofluorescence with Shh (red) shows expression in the cloaca epithelium in E11.5 WT embryo (E) while absent in the KO (F). (G-H) WT cloaca epithelium expresses Keratin 7 (red) at the distal part of the cloaca and Keratin 8 (green) throughout the epithelium (G) in contrast to the KO where Keratin 8 is partially expressed in the epithelium as denoted by the white arrows (H). Scale bars: 100 µm (A,D), 50 µm (B-C), 20 µm (E-H). Abbreviations: cl: cloaca, hg: hindgut, ur: urethra, ugs: urogenital sinus, ec: ectoderm;

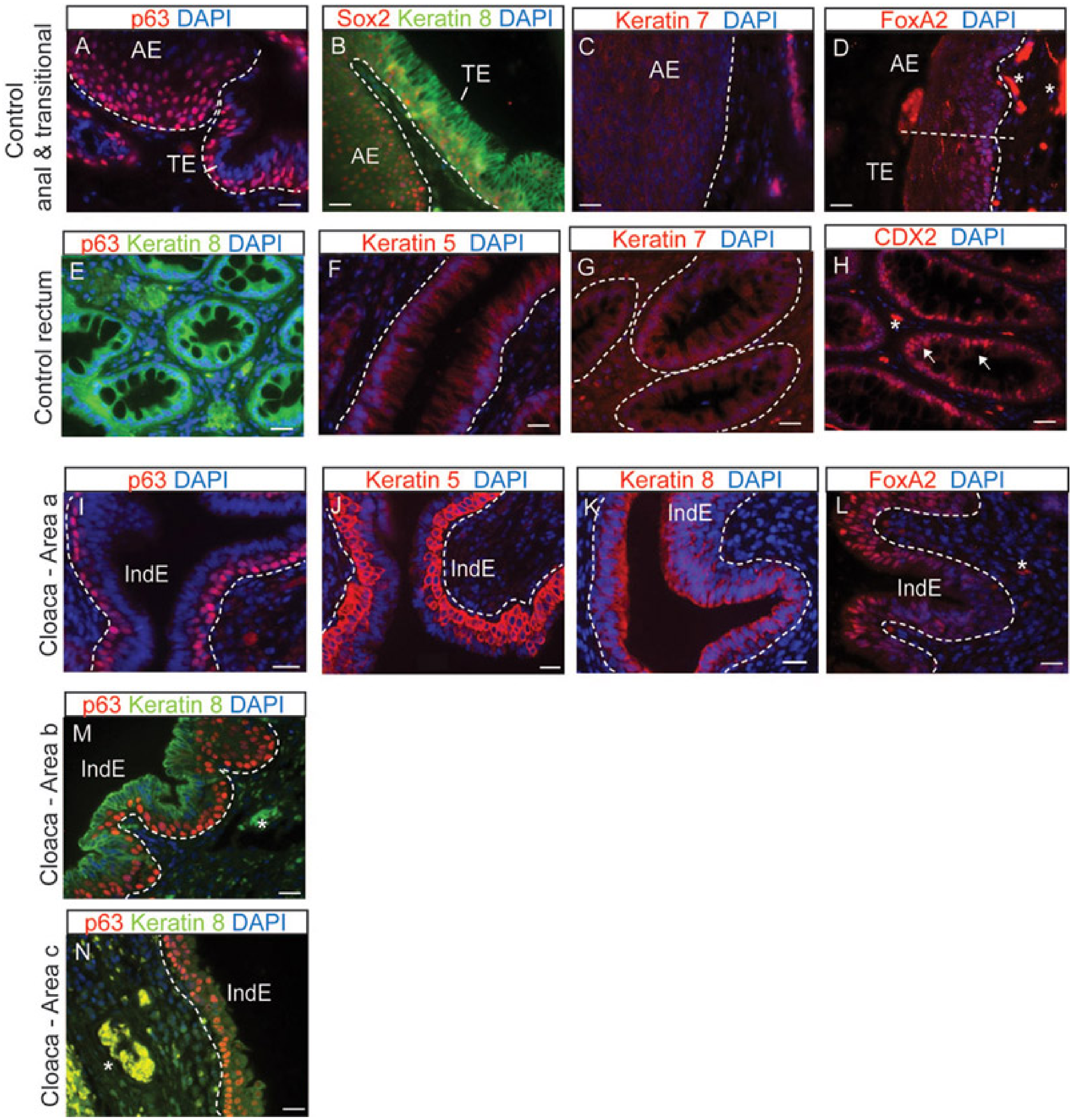

Fig. S2: Molecular identity of the indeterminate epithelium in human cloaca patients. (A-N) Immunofluorescence analysis for the indicated markers in control anal and transitional epithelium (A-D), control rectum (E-H), cloaca from area a (I-L), cloaca from area b (M) and cloaca from area c (N). (A-D) Markers of stratified squamous and transitional epithelia include p63 (red) (A), and Sox2 (red) (B) are all expressed in the anal canal and transitional epithelium of normal tissue from an idiopathic prolapse sample. Keratin 8 (green) marks the transitional epithelium (B). Keratin 7 (red) and FoxA2 (red) are not expressed in the anal epithelium (C and D). (E-H) Control rectum expresses Keratin 8 (green) (E) and the hindgut marker CDX2 (H) and does not express p63 (E), Keratin 5 (F) and Keratin 7 (G). (I-L) The indeterminate epithelium in cloaca from area a expresses p63 (I), Keratin 5 (J), Keratin 8 (K) and FoxA2 (L). (M) The indeterminate epithelium in cloaca from area b is more specified than the cloaca from the region c as denoted by the expression of Keratin 8 (green) in the suprabasal layer of the epithelium and the expression of p63 (red) in the basal cells. (N) The indeterminate epithelium present in cloaca from area c expresses markers of stratified squamous epithelia such as p63 (red) and does not express Keratin 8 (green) marking simple epithelia. The dotted lines mark the epithelia. Abbreviations: AE: Anal epithelium; TE: Transitional epithelium, IndE: Indeterminate epithelium. The asterisk denotes autofluorescence. All scale bars: 20 μm.

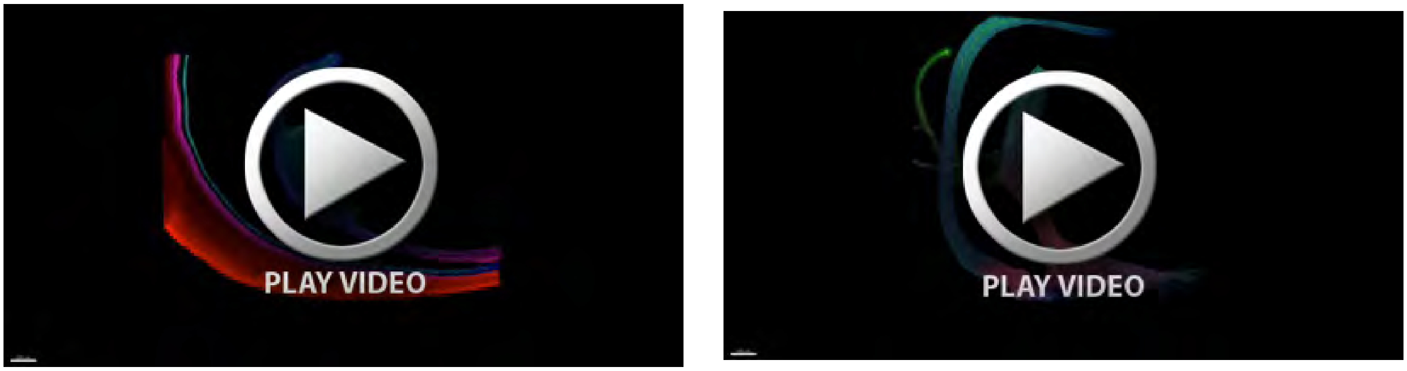

Movie 1. Early patterning of the cloaca epithelium.

Whole mount immunostaining of E10.5 WT (movie 1A) and E13.5 WT (movie 1B) embryos with the indicated markers: FoxA2 (blue), Keratin 8 (green) and Sox2 (red). At E10.5, a population of cells positive for Sox2 is restricted to the dorsal side of the cloaca epithelium. At E13.5 when septation is complete, Sox2 positive cells are found in the anal canal and urethra.

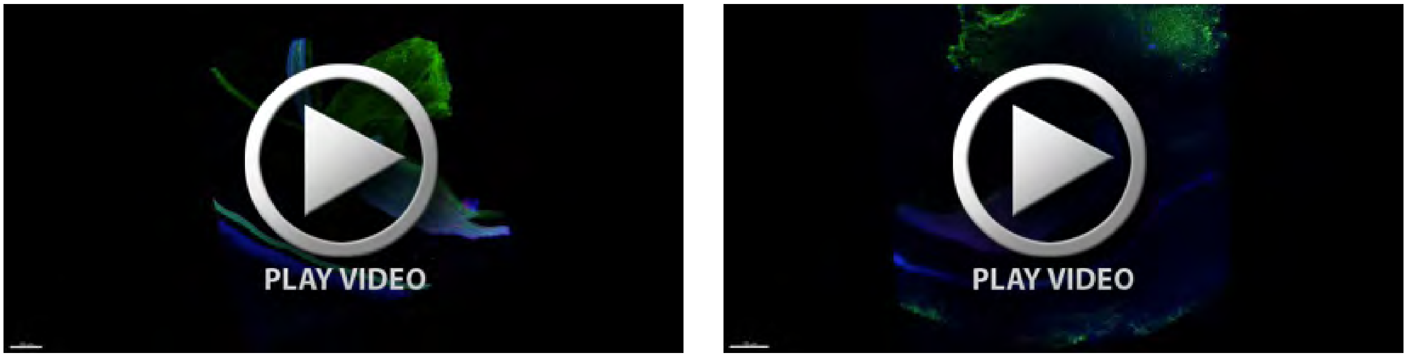

Movie 2. Defect in the cloaca epithelium of *Shh* knockout mice.

Whole mount immunostaining of E11.5 WT (movie 2A) and *Shh* knockout (movie 2B) embryos with the indicated markers: FoxA2 (blue), Keratin 8 (green) and Sox2 (red). While Keratin 8 is highly expressed in the WT cloaca epithelium, it is markedly decreased in the knockout. Sox2 is also reduced in the knockout epithelium. Stainings have been done on at least 3 WT and 3 knockout and a representative example is shown.

Runck et al., Table S1

		Tissue-type	Shh expression	p-Smad1-5-8 expression
**Control vagina**		Vagina	pos basal layer epithelium, some in stroma	pos basal layer epithelium, some in stroma
**Control anorectum**		Anal canal		pos basal layer epithelium
		Transitional epithelium	pos crypt	pos crypt and transitional epithelium
**Perineal Fistula**		Rectum	pos in some crypt cells, neg in stroma	pos in anal-type epithelium
		Transitional epithelium	weak/neg epithelium	pos transitional epithelium, pos stroma
**Cloaca**				
**Case**	**Area**			
**1**	**a**	Vagina	weak basal layer, weak/neg stroma	neg vagina, neg stroma
		Rectum	weak crypt, pos in stroma	
		Distal Indeterminate	pos epithelia, pos in stroma	
		Proximal Indeterminate	neg/weak epithelia, weak stroma	neg indeterminate, neg stroma
**2**	**a**	Vagina	weak/neg epithelium, neg stroma	
		Indeterminate	weak epithelium, pos in stroma	
		Distal Rectum	pos crypt, pos in stroma	pos/weak indeterminate
		Proximal Rectum	neg epithelia, neg stroma	neg rectum
**3**	**a**	Vagina	neg basal layer, neg stroma	neg vagina, neg stroma
**4**	**a**	Rectum	weak/neg crypt, neg stroma	neg indeterminate and rectum epithelium
**5**	**b**	Rectum	few pos crypt cells, neg in stroma	pos indeterminate
**6**	**b**	Indeterminate	neg epithelia, neg stroma	neg epithelia, neg stroma
		Proximal Rectum	neg crypt, neg stroma	neg epithelia, neg stroma
**7**	**b**	Indeterminate	neg epithelia, neg stroma	neg epithelia, neg stroma
**8**	**b**	Indeterminate	neg epithelia, neg stroma	neg epithelia, neg stroma
**9**	**b**	Indeterminate	neg epithelia, neg stroma	neg epithelia, neg stroma
**10**	**c**	Indeterminate	neg epithelia, neg stroma	neg epithelia, neg stroma
**11**	**c**	Indeterminate	neg epithelia, neg stroma	pos epithelia, neg stroma
**12**	**c**	Indeterminate	neg epithelia, neg stroma	neg epithelia, neg stroma
**13**	**c**	Indeterminate	neg epithelia, neg stroma	neg epithelia, neg stroma
**14**	**c**	Indeterminate	neg epithelia, neg stroma	neg epithelia, neg stroma

Table S1. Summary of Shh and p-Smad1/5/8 expression analyzed by immunofluorescence on human tissues. Human tissues include control vagina, control anorectum from an idiopathic prolapse sample, perineal fistula, and 14 cloaca patients from region a, b and c (see Fig. 2B for the location of each biopsy). Shh and p-Smad expression has been reported for each tissue-type. Abbreviations: pos: positive, neg: negative.
